# Attention effects on the processing of task-relevant and task-irrelevant speech sounds and letters

**DOI:** 10.3389/fnins.2013.00231

**Published:** 2013-12-03

**Authors:** Maria Mittag, Karina Inauri, Tatu Huovilainen, Miika Leminen, Emma Salo, Teemu Rinne, Teija Kujala, Kimmo Alho

**Affiliations:** ^1^Cognitive Brain Research Unit, Cognitive Science, Institute of Behavioural Sciences, University of HelsinkiHelsinki, Finland; ^2^Division of Cognitive Psychology and Neuropsychology, Institute of Behavioural Sciences, University of HelsinkiHelsinki, Finland; ^3^Finnish Centre of Excellence in Interdisciplinary Music Research, University of JyväskyläJyväskylä, Finland; ^4^Cicero Learning Network, University of HelsinkiHelsinki, Finland; ^5^Helsinki Collegium for Advanced Studies, University of HelsinkiHelsinki, Finland

**Keywords:** attention, suppression, event-related potential, EEG, audition, speech

## Abstract

We used event-related brain potentials (ERPs) to study effects of selective attention on the processing of attended and unattended spoken syllables and letters. Participants were presented with syllables randomly occurring in the left or right ear and spoken by different voices and with a concurrent foveal stream of consonant letters written in darker or lighter fonts. During auditory phonological (A_P_) and non-phonological tasks, they responded to syllables in a designated ear starting with a vowel and spoken by female voices, respectively. These syllables occurred infrequently among standard syllables starting with a consonant and spoken by male voices. During visual phonological and non-phonological tasks, they responded to consonant letters with names starting with a vowel and to letters written in dark fonts, respectively. These letters occurred infrequently among standard letters with names starting with a consonant and written in light fonts. To examine genuine effects of attention and task on ERPs not overlapped by ERPs associated with target processing or deviance detection, these effects were studied only in ERPs to auditory and visual standards. During selective listening to syllables in a designated ear, ERPs to the attended syllables were negatively displaced during both phonological and non-phonological auditory tasks. Selective attention to letters elicited an early negative displacement and a subsequent positive displacement (Pd) of ERPs to attended letters being larger during the visual phonological than non-phonological task suggesting a higher demand for attention during the visual phonological task. Active suppression of unattended speech during the A_P_ and non-phonological tasks and during the visual phonological tasks was suggested by a rejection positivity (RP) to unattended syllables. We also found evidence for suppression of the processing of task-irrelevant visual stimuli in visual ERPs during auditory tasks involving left-ear syllables.

## Introduction

In everyday situations, our sensory systems receive much more information than we can consciously and actively process. However, selective attention enables us to focus on task-relevant stimuli and to largely ignore task-irrelevant information (Pashler, 1997). most previous event-related potential (ERP) studies on auditory selective attention examined the effects of attention on the processing of simple tones during auditory non-linguistic tasks (e.g., Hillyard et al., 1973; Näätänen et al., 1978; Hari et al., 1989; Woldorff et al., 1993) or the processing of speech sounds during auditory linguistic tasks (e.g., Woods et al., 1984; Teder et al., 1993). Moreover, there are studies suggesting that in addition to enhancing processing of attended tones, selective attention may suppress processing of ignored tones (e.g., Alho et al., 1987, 1994; Berman et al., 1989; Michie et al., 1990; Woods, 1990; Degerman et al., 2008) and at least one study reporting similar selective-attention effects on event-related brain potentials (ERPs) to tones during two different tasks (counting silently all attended tones vs. counting infrequent target tones among the attended tones; Alho et al., 1990). However, to our knowledge, there are no previous ERP studies focusing on the effects of selective attention and task type on the processing of attended and ignored speech. Therefore, in the present ERP study, we compared effects of selective attention on the processing of attended and ignored spoken syllables during auditory phonological (A_P_) and non-phonological tasks. In addition, we compared the processing of ignored spoken syllables during visual phonological and non-phonological tasks. Since the visual stimuli attended during the visual tasks and ignored during the auditory tasks were written letters, the present experiment allowed us also to study possible effects of auditory and visual attention and tasks on the processing of visually presented linguistic material.

Previous research (e.g., Hillyard et al., 1973; Näätänen et al., 1978; Hari et al., 1989; Rif et al., 1991; Woldorff et al., 1993) has shown that strongly focused selective attention modulates ERPs and event-related magnetic fields (ERFs measured with magnetoencephalography) to tones or speech sounds within the first 100 ms from the sound onset. These studies have shown, for example, an effect of selective attention on the N1 ERP/ERF response which has bilateral auditory cortex generators and a negative-polarity maximum over fronto-central scalp areas around 100 ms from sound onset: when concurrent rapid sequences of tones are delivered to the opposite ears, the N1 is elicited with a larger amplitude by attended tones delivered to one ear than by the same tones when they are ignored and tones delivered to the other ear are attended in turn (Hillyard et al., 1973; Rif et al., 1991; Woldorff et al., 1993). This N1 effect is often overlapped and/or followed by the processing negativity (PN) ERP response specifically associated with selective auditory attention (e.g., Näätänen et al., 1978, 1992; Alho, 1992). The PN usually causes a long-duration negative displacement (called the negative difference, Nd) of ERPs to the attended tones in relation to ERPs to unattended tones (e.g., Näätänen et al., 1978; Hansen and Hillyard, 1980; Hari et al., 1989; Alho, 1992; Degerman et al., 2008).

In addition to an N1 enhancement and PN indicating preferential processing of the attended sounds, some studies suggested that the Nd might be partly due to a rejection positivity (RP) elicited by unattended sounds around 200–300 ms from their onset (Alho et al., 1987, 1994; Berman et al., 1989; Michie et al., 1990; Degerman et al., 2008). In these studies, the RP was revealed by comparing ERPs to unattended sounds during selective listening to other sounds with ERPs to the same sounds when ignored during attention to visual stimuli. The RP was suggested to be associated with active suppression of the processing of unattended sounds.

Moreover, the Nd usually has also a later portion that starts around 300 ms from sound onset with a frontally dominant distribution. The late Nd has been suggested to be associated with further processing of the selectively attended sounds or with prefrontal functions maintaining the selective state (the so-called attentional trace) in the auditory cortex (Näätänen, 1982, 1990; Giard et al., 1988; Alho et al., 1992, 1994). MEG recordings showed that even the late Nd gets a contribution from the auditory cortex (Hari et al., 1989; Degerman et al., 2008). This supports the view that further processing of the attended sounds contributes to the late portion of Nd.

Nd effects are also elicited by spoken syllables and words during selective listening (Hansen et al., 1983; Woods et al., 1984; Teder et al., 1993). Moreover, the processing of competing speech sounds synchronously delivered to the opposite ears has been studied in behavioral dichotic listening experiments. In such condition, listeners primarily perceived sounds delivered to the right ear. This so-called right-ear advantage (REA) has been interpreted to be associated with a bias of auditory attention to the right (Kinsbourne, 1970; Takio et al., 2011) or even with a more general, multimodal rightward bias of attention (Hämäläinen and Takio, 2010). Alternatively, it has been suggested that REA for speech occurs because right-ear speech inputs reach left-hemisphere speech processing areas faster than left-ear inputs (Kimura, 1967; Hugdahl, 2003). REA during dichotic listening was supported by our recent MEG study (Alho et al., 2012). In that study, we observed a stronger sustained ERF at 300–500 ms in the auditory cortex contralateral to the attended direction than in the ipsilateral auditory cortex during selective listening to the right-ear or left-ear syllables in dichotic syllable pairs. Further, in a condition with binaural or “non-forced” attention, the listeners showed a classic REA by detecting more target syllables delivered to the right ear than target syllables delivered to the left ear. Like during selective listening to the right-ear syllables, the sustained ERF was stronger in the left auditory cortex than in the right auditory cortex during non-forced attention. This suggests that the REA observed in the latter condition was due to bias of auditory attention to the right.

Suppression of the processing of task-irrelevant speech sounds, in turn, was suggested by our recent functional magnetic resonance imaging (fMRI) study (Salo et al., 2013). In this study, participants selectively attended to independent streams of spoken syllables and written letters and performed a “simple” (speaker-gender or font-shade), spatial or phonological discrimination task involving the attended stimuli. While activity in the superior temporal sulcus (STS) was enhanced by all auditory tasks in relation to all visual tasks, STS activity to unattended speech sounds was decreased during the visual phonological task in relation to visual non-phonological tasks (see also Crottaz-Herbette et al., 2004). This effect for the visual phonological task suggested enhanced demand for suppressing the processing of task-irrelevant, distracting spoken syllables especially during this task (Salamé and Baddeley, 1982; Salo et al., 2013). Support for the notion that the suppression effect in the STS is specific for phonological processing of visual inputs (Salo et al., 2013) is given by another fMRI study by Rinne (2010). He found that auditory-cortex activations to non-speech sounds (noise bursts with pitch) were only slightly and non-significantly attenuated with an increased difficulty in a visual discrimination task involving non-linguistic stimuli.

Inspired by the suppression effect found for unattended speech in our previous study (Salo et al., 2013), we investigated in the present study whether ERPs to task-irrelevant spoken syllables during a visual phonological task would show an RP in relation to ERPs to task-irrelevant spoken syllables during a visual non-phonological task. In addition, we compared Nd and RP effects in ERPs to attended and unattended spoken syllables, respectively, during A_P_ and non-phonological tasks. We used a quite similar experimental setup as in our previous fMRI study (Salo et al., 2013) except that we omitted auditory and visual spatial discrimination tasks. We presented the participants of the present study with spoken syllables randomly to the left and right ear and with a concurrent stream of written letters. In different conditions, the participants were instructed to attend to the left-ear syllables, right-ear syllables, or written letters and to perform a phonological or non-phonological (speaker-gender or font-shade) discrimination task involving the attended stimuli. In addition to the processing of attended and unattended spoken syllables during phonological and non-phonological auditory and visual tasks, the present experimental paradigm allowed us to investigate effects of attention on ERPs to written letters during visual phonological and non-phonological tasks. Also, we could investigate possible modulation of the processing of written letters during A_P_ and non-phonological tasks involving the left-ear or right-ear syllables.

Our hypotheses were as follows. (1) We expected to find a negative displacement of ERPs to selectively attended spoken syllables in relation to ERPs to unattended syllables in accordance with previous studies on selective listening to tones or speech sounds (e.g., Hillyard et al., 1973; Näätänen et al., 1978; Woods et al., 1984; Teder et al., 1993; Alho et al., 1994). (2) Since previous studies found support for active suppression of unattended tones (Alho et al., 1987, 1994; Berman et al., 1989; Michie et al., 1990; Degerman et al., 2008), we expected to find evidence for suppression of ignored spoken syllables, that is, we expected that ERPs to syllables delivered to one ear would be positively displaced when they are ignored and syllables delivered to the other ear are attended in relation to ERPs to the same syllables during the visual non-phonological task. (3) As a new finding, we expected to observe RPs in ERPs to unattended spoken syllables during the visual phonological task in relation to ERPs to spoken syllables during the visual non-phonological task, since our previous fMRI results showed suppression of the processing of irrelevant speech during phonological processing of written letters (Salo et al., 2013). (4) Based on previous results on visual attention effects (e.g., Hillyard and Anllo-Vento, 1998; Salmi et al., 2007), we expected to find a visual Nd, possible followed by a positive displacement (Pd), in the ERPs to letters attended during the visual tasks in relation to ERPs to letters ignored during the auditory tasks. In addition to testing these hypothesis, we explored ERP signs of suppression of the processing of unattended written letters during the auditory tasks.

## Methods

### Participants

Thirty-one right-handed native Finnish speakers with self-reported normal hearing, normal or corrected-to-normal vision and without history of neurological or mental disease participated in the study. All participants were volunteers, gave written informed consent, and received cultural vouchers or study credits for their participation in the experiment. Data from one participant was discarded because the participant wanted to quit participation before the experiment was completed. In addition, data from two participants were excluded due to extensive EEG alpha activity, and from two other participants because of technical problems during data collection. Hence, data from 26 participants (20–43 years old; 11 males) are reported here. The study protocol was approved by the University of Helsinki Ethical Review Board in the Humanities and Social and Behavioral Sciences.

### Stimuli

Auditory stimuli were eight meaningless syllables chosen from the set of syllables used in our previous study (Salo et al., 2013). Four of them were consonant-vowel syllables (/ku/, /lu/, /mu/, /pu/) and the other four were vowel-consonant syllables (/ah/, /ak/, /ap/, /at/). The syllables were spoken by four female and four male native Finnish speaking young adults and recorded with a t.bone SC440 microphone (Bund International Ltd., Ningbo, China). The intensity of the syllables was normalized between the speakers and the duration of each syllable was cut to 250 ms with linear 2-ms rise and 5-ms fall times using Adobe Audition 3 (Adobe Systems Inc., San Jose, California, USA). The syllables were delivered monaurally through headphones (MDR-7506 Professional, Sony Corp., Tokyo, Japan) at an intensity of 50 dB above individual hearing threshold.

Visual stimuli were eight written consonant letters used in our previous study (Salo et al., 2013). The Finnish names of four of these letters (L, M, R, S) started with a vowel (for example, in English, the name of letter R is pronounced like “are” and thus starts with a vowel) the names of four other letters (C, P, T, V) started with a consonant (for example, in English, the name of letter T is pronounced like “tea” and thus ends in a vowel). Each letter was presented in uppercase Arial font for 250 ms with a visual angle of 0.77° (19″ Dell 1908 FPb, Dell Inc., USA, refresh frequency of 75 Hz). The letters were presented in the center of the screen on a gray background (RGB values of 127 each) located in front of the participant at a distance of 1.5 m. The letters were presented in gray fonts, four of them darker than the background (R, G, and B either 16, 32, 48, or 64 each) or four of them lighter than the background (R, G, and B either 192, 208, 224, or 240 each).

### Experimental procedure

**Figure 1:**
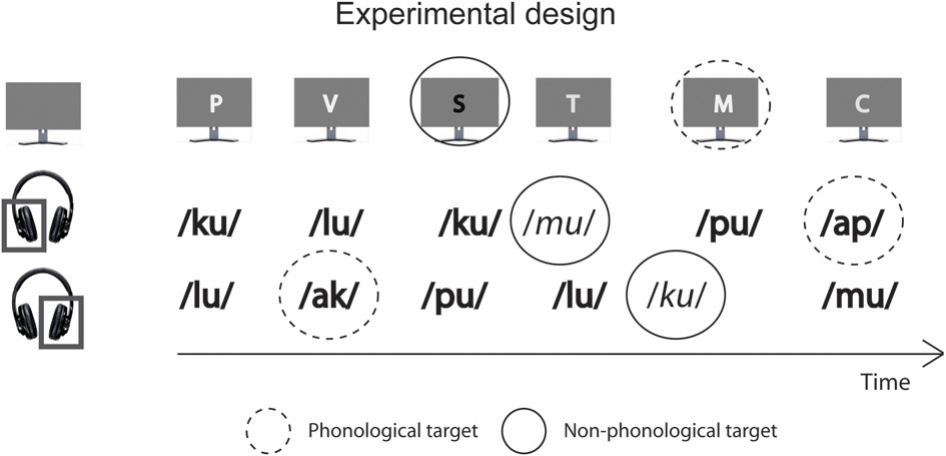
**A schematic illustration of experimental conditions.** In all conditions, the participants were presented with spoken syllables and written letters. They were instructed to selectively attend to syllables delivered to the left ear, to syllables delivered to the right ear, or to the written letters and to perform a phonological or non-phonological task involving the attended stimuli. Most syllables were spoken by one of the four male voices (syllables printed in bold) and started with a consonant. In the left-ear and right-ear phonological tasks (L_P_ and R_P_, respectively), targets were infrequent attended-ear syllables starting with a vowel (marked with a dashed circle), and in the left-ear and right-ear non-phonological tasks (L_NP_ and R_NP_, respectively), targets were infrequent syllables spoken by one of the four female voices (syllables printed in italic and marked with a continuous circle). Most visually presented consonant letters had a name starting with a consonant (e.g., the English name of the letter T is pronounced like “tea” and thus starts with a consonant) and were written in one of four fonts lighter than the gray background on the screen. In the visual phonological and non-phonological tasks (V_P_ and V_NP_, respectively), targets were infrequent letters with their name beginning with a vowel (marked with a dashed circle; e.g., the English name of letter R is pronounced like “are” and thus starts with a vowel) or infrequent letters written in one of four fonts that were darker than the background (marked with a continuous circle).

The experimental design is illustrated in Figure 1. Each stimulus block included independent sequences of auditory and visual stimuli and was 120 s long. In the auditory sequences, 240 syllables in a random order to the left and right ear (120 syllables to each ear) with stimulus onset asynchronies (SOAs) varying randomly between 400 and 600 ms in 10-ms steps with an even distribution. Therefore, syllables presented to the left and right ear never overlapped in time. For each ear, there were 72 “standard” syllables spoken by a male voice and ending in a vowel (standard syllables), 24 “voice-deviant” syllables spoken by a female voice and ending in a vowel, and 24 “phonologically deviant” syllables spoken by a male voice and starting with a vowel delivered in a random order except that within each ear, a voice deviant or phonologically deviant syllable was always followed by a standard syllable. In each of the three syllable categories, the four different voices, and the four different syllables occurred in a random order.

The visual stimulus sequences contained 120 stimuli delivered with SOAs varying randomly between 400 and 1600 ms in steps of 100 ms with an even distribution. The visual and auditory sequences had independent SOAs, with the restriction that their onsets never overlapped in time. Each sequence contained 72 “standard” letters written in lighter-than-background font and with their name starting with a consonant, 24 “font-deviant” letters written in darker-than-background font and with their name starting with a consonant, and 24 “phonologically deviant” letters written in lighter-than-background font and with their name starting with a vowel of the present study delivered in a random order except that a font-shade deviant letter or a phonologically deviant letter was always followed by a standard letter. In each of the three visual stimulus categories, the four different font shades, and the four different letters occurred in a random order.

There were six different experimental conditions: phonological and non-phonological tasks for the left-ear syllables (L_P_ and L_NP_, respectively), phonological and non-phonological tasks for the right-ear syllables (R_P_ and R_NP_, respectively), and phonological and non-phonological tasks for the visually presented letters (V_P_ and V_NP_, respectively). In the L_P_ and L_NP_ tasks, targets were left-ear phonologically deviant and voice deviant syllables, respectively. In the R_P_ and R_NP_ tasks, targets were right-ear phonologically deviant and voice deviant stimuli, respectively. In the V_P_ and V_NP_ tasks, targets were phonologically deviant and font-shade deviant letters, respectively. For each of the six conditions there were six stimulus blocks. Thus, the experiment consisted of 36 blocks with the experimental task varying in an order randomized separately for each participant. Before each block, a written task instruction in Finnish was given in the middle of the screen. In each condition, the participants were required to focus on the center of the screen and to press a button with their right thumb to the designated targets (response button box: RB-834 by Cedrus Corporation, USA). They were also asked to keep eye movements, blinking and head movements to a minimum during the tasks. A short practice run was performed before the actual experiment to ensure that the participants felt comfortable undertaking each task. Stimuli were delivered using Presentation 14.9.07.19.11 software (Neurobehavioral Systems, Inc., Albany, CA, USA).

### Analysis of behavioral data

Button presses given within 200–1000 ms from target stimulus onset were classified as hits and mean reaction times (RTs) were calculated for them. Hit rates (HRs) were calculated as the number of hits divided by the number of targets. All other button presses were regarded as false alarms. False-alarm rates (FaRs) were calculated as the number of false-alarms divided by the number of all non-target stimuli in the attended stream (the left-ear, right-ear or visual stream). All three performance measures (RTs, HRs, FaRs) were analyzed separately by using two repeated-measures analyses of variance (ANOVAs), one to compare performance in the auditory tasks (data for the left-ear and right-ear tasks combined) and visual tasks with factors Modality (Audition, Vision) and Task (Phonological, Non-Phonological), and the other to compare performance in the left-ear and right-ear tasks with factors Ear (Left, Right) and Task (Phonological, Non-Phonological). The ANOVAs that showed significant (*p* < 0.05) main effects of factors or their interactions were followed by Bonferroni *post-hoc* tests for pairwise comparisons of different tasks.

### EEG recording and ERP averaging

The experiment was carried out in an electrically and acoustically shielded room. EEG (bandwidth from DC to 104 Hz and sampling rate of 512 Hz) was recorded with 64 active scalp electrodes (BioSemi ActiveTwo System and ActiView605-Lores, BioSemi B.V., Amsterdam, the Netherlands) and additional electrodes on the left and right mastoids. All EEG electrodes were online referenced to Common Mode Sense (CMS) electrode with standard location at PO1. Vertical electro-oculography (EOG) was recorded with an electrode placed below the left eye. Horizontal EOG was recorded with electrodes placed near the canthus of each eye. EEG was re-referenced offline to the averaged recordings from the left and right mastoids.

Continuous EEG data were digitally offline filtered with 0.5 Hz high-pass and 30 Hz low-pass filters. ERPs were obtained separately for each stimulus type and condition by averaging EEG epochs starting 100 ms before each stimulus onset and ending 700 ms after each onset. The mean voltage over the 100-ms pre-stimulus period was used as the 0-μV baseline. For each experimental block, the first five stimuli were excluded from averaging. Eye-movement and blink artifacts were automatically corrected by using a spatial filtering algorithm [adaptive artifact correction; Ille et al. (2002)] in Besa 5.3 software (Besa Software GmbH, Gräfelfing, Germany). EEG epochs with peak-to-peak amplitudes exceeding 150 μ V after this correction were excluded from averaging. For one participant, data from one EEG channel with a poor contact were replaced with data interpolated from neighboring channels with BESA spherical spline interpolation method. In different conditions, the number of accepted epochs ranged from 302 to 432 for each type of standard stimulus (left-ear, right-ear, and visual standards) and from 101 to 144 for each type of deviant stimuli (left-ear, right-ear, and visual phonological and non-phonological deviants). Thus, at least 70% of all epochs were acceptable in each participant.

### ERP analysis

Only ERPs to the auditory and visual standard stimuli will be reported here. ERPs to deviant stimuli were not analyzed further because of three reasons: (1) For each condition, the number of certain deviant stimuli was relatively small leading to less reliable ERPs to deviants; (2) there were systematic physical differences between standard and deviant stimuli (except the visual phonological deviants) and therefore there were also systematic differences in the exogenous ERP responses (e.g., the auditory N1 response) to the deviants and standards; and (3) the ERPs to the deviant target stimuli presumably included contributions from movement-related activity (e.g., Knight et al., 1989) complicating comparison of target ERPs with ERPs to non-target deviants and with ERPs to standards.

#### Analysis of ERPs to standard syllables

Overall effects of selective auditory attention (*negative difference waves*, Nds, Hansen and Hillyard, 1980) on ERPs to the left-ear standard syllables were investigated by subtracting ERPs to the unattended left-ear standard syllables during the R_P_ and R_NP_ tasks from ERPs to the attended left-ear standard syllables during the L_P_ and L_NP_ tasks, respectively. Effects of selective auditory attention on ERPs to the right-ear standard syllables were determined accordingly. Furthermore, we investigated suppression of the processing of syllables delivered to the unattended ear during attention to the other ear by means of *suppression difference waves*. To this end, we subtracted ERPs to the unattended left-ear standard syllables during the V_NP_ from ERPs to the unattended left-ear standard syllables during the R_P_ and R_NP_ (cf. Alho et al., 1994). Suppression effects for unattended right-ear standard syllables were calculated accordingly. In the subtractions, we used the V_NP_ condition as the baseline, since we expected suppression of the processing of the unattended left-ear or right-ear syllables also during the V_P_ task (cf. Salo et al., 2013). This expected suppression was examined by means of suppression difference waves obtained by subtracting ERPs to the left-ear and right-ear standard syllables during the V_NP_ task from ERPs to the left-ear and right-ear standard syllables, respectively, during the V_P_ task.

To study the expected early-Nd effect, amplitudes of the ERP difference waveforms between 50 and 300 ms were measured as mean amplitudes over consecutive 50-ms time windows. For each difference wave and time window, the significance of difference-wave amplitude was tested with one-tailed *t*-tests (Bonferroni-corrected for *t*-tests at 5 time windows). To study the expected late-Nd effects, amplitudes of the ERP difference waveform amplitudes between from 300 to 700 ms were measured as mean amplitudes over consecutive 100-ms time windows. For each difference wave and time window, the significance of difference-wave amplitude was tested with one-tailed *t*-tests (Bonferroni-corrected for *t*-tests at 4 time windows).

The early and late Nds were further analyzed with Four-Way repeated-measures ANOVAs with factors Task (Phonological, Non-Phonological), Stimulated Ear (Left, Right), Frontality, and Laterality. The factor Frontality had 5 levels: Frontal (electrodes F5, F3, F1, Fz, F2, F4, and F6), Fronto-central (FC5, FC3, FC1, FCz, FC2, FC4, and FC6), Central (C5, C3, C1, Cz, C2, C4, and C6), Centro-parietal (CP5, CP3, CP1, CPz, CP2, CP4, and CP6), and Parietal (P5, P3, P1, Pz, P2, P4, and P6) and the factor Laterality had 7 levels: Lateral Left (F5, FC5, C5, CP5, and P5), Middle Left (F3, FC3, C3, CP3, and P3), Medial Left (F1, FC1, C1, CP1, and P1), Central (Fz, FCz, Cz, CPz, and Pz), Medial Right (F2, FC2, C2, CP2, and P2), Middle Right (F4, FC4, C4, CP4, and P4), and Lateral Right (F6, FC6, C6, CP6, and P6).

Amplitudes of the expected suppression effects on ERPs to spoken syllables during attention to the opposite-ear syllables or during the V_P_ task, in turn, were measured from the suppression difference waves over consecutive 50-ms periods between 150 and 300 ms. For each difference wave and time window, the significance of difference-wave amplitude was tested with one-tailed *t*-tests (Bonferroni-corrected for *t*-tests at 3 time windows). We conducted similar Four-Way repeated-measures ANOVA for the amplitudes of suppression difference waves as for the early and late Nd. For examining the expected suppression effects of the V_P_ task on the processing of unattended spoken syllables, we conducted a Three-Way repeated-measures ANOVA for the suppression difference wave amplitudes with factors Ear, Frontality, and Laterality. Furthermore, the suppression effects on the processing of unattended spoken syllables during auditory and visual phonological tasks were compared by using the same set of 35 electrodes, with the factors Attended Modality (Audition, Vision), Stimulated Ear (Left, Right), Frontality, and Laterality.

#### Analysis of ERPs to standard letters

Based on our previous study on effects of attention on ERPs to auditory and visual stimuli (Salmi et al., 2007) including stimuli in central space delivered at rates comparable to the present stimuli, we expected to see an Nd effect of attention also in the visual ERPs. This effect, shown as a negative displacement of ERPs to the written letters during visual attention in relation to ERPs to the written letters during auditory attention, was expected to occur around 150–300 ms from stimulus onset. In addition, we expected a subsequent Pd effect around 300–700 ms, that is, a positive displacement of the ERPs to written letters during visual attention in relation to the ERPs to written letters during auditory attention (see also, Hillyard and Anllo-Vento, 1998).

Therefore, we studied the effects of attention on written letters by comparing the ERPs to written letters during the V_P_ and V_NP_ tasks with the ERPs to written letters during the A_P_ and non-phonological tasks. To evaluate these effects, ERPs to the standard letters during the A_P_ tasks (data collapsed across the L_P_ and R_P_ conditions) were subtracted from ERPs to the standard letters during the V_P_ task. The difference waves for the non-phonological tasks were calculated accordingly. Furthermore, we compared the visual ERPs (N1 and P2 components, Heinze et al., 1990; Luck et al., 1990) during the four auditory tasks (L_P_, L_NP_, R_P_, and R_NP_) in order to see possible effects of auditory task or direction of auditory attention on early visual processing at 50–300 ms.

To investigate the expected early Nd effect of selective auditory attention on the processing of written letters, mean amplitudes of the visual ERP difference waveforms between 150 and 300 ms were measured over consecutive 50-ms time windows. For each difference wave and time window, the significance was tested with one-tailed *t*-tests (Bonferroni corrected for *t*-tests at 3 time windows). To study the expected later Pd effects, mean amplitudes of the visual ERP difference waveform were calculated over consecutive 100-ms time windows between 300 and 600 ms. The significance of difference-wave amplitude was tested with one-tailed *t*-tests (Bonferroni corrected for *t*-tests at 3 time windows) for each difference wave and time window. For both Nd and Pd effects, we conducted Three-Way repeated-measures ANOVAs with the factor Task (Phonological, Non-Phonological) on the ERP amplitudes in an array of 18 electrodes categorized by factors: Frontality [6 levels: Fronto-central (FC3, FCz, and FC4), Central (C3, Cz, and C4), Centro-parietal (CP3, CPz, and CP4), and Parietal (P3, Pz, and P4), Parieto-Occipital (PO3, POz, and PO4) and Occipital (O1, Oz, and O2)] and Laterality [3 levels: Left (FC3, C3, CP3, P3, PO3, and O1), Central (FCz, Cz, CPz, Pz, POz, and Oz), and Right (FC4, C4, CP4, P4, PO4, and O2)].

Finally, since we had no exact hypothesis on possible effects of lateral auditory attention on processing unattended foveal letters due to the lack of previous research, we measured the amplitudes of the visual N1 and P2 responses as mean amplitudes over 100–150 and 200–300 ms from letter appearance. This was done separately for the four auditory tasks (L_P_, L_NP_, R_P_, and R_NP_) at O1 and O2 electrodes over the left and right visual cortex, respectively. We conducted separate repeated-measures ANOVAs for the N1 and P2 amplitudes with the factors: Attended Ear (Left, Right), Auditory Task (Phonological, Non-Phonological), and Electrode (O1, O2).

In all ANOVAs, Greenhouse-Geisser corrections were applied when needed (in these cases the correction value ε is given). However, even for these ANOVAs, *p*-values will be reported with the original degrees of freedom. The ANOVAs showing significant (*p* < 0.05) main effects of factors or their interactions were followed by Bonferroni *post-hoc* tests for pairwise comparisons of different tasks.

## Results

### Performance

**Figure 2:**
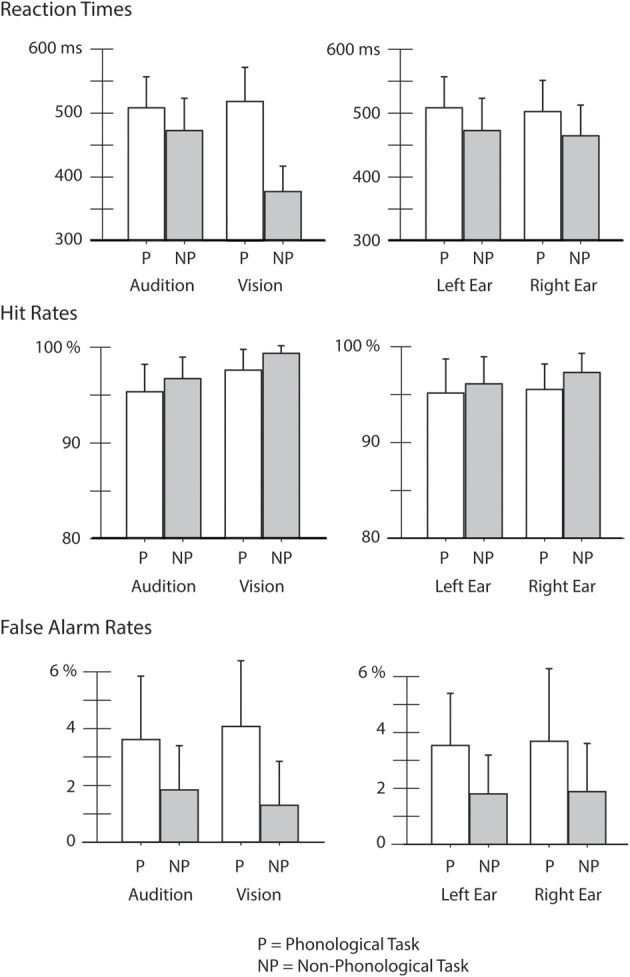
**Reaction times (RTs), hit rates (HRs), and false-alarm rates (FaRs) in different experimental conditions.** Performance is compared between the auditory and visual tasks (Audition vs. Vision) while participants performed a phonological (P) or non-phonological task (NP) in the attended modality (for Audition data for the left-ear and right-ear tasks were combined) and within the auditory domain between the left-ear and right-ear tasks while participants performed an auditory phonological task (P) and an auditory non-phonological task (NP).

Mean RTs, HRs, and FaRs are shown in Figure 2. Comparison of RTs in the auditory and visual tasks with an ANOVA showed a significant Modality × Task interaction [*F*_(1, 25)_ = 1.2, *p* < 0.001, η^2^_*p*_ = 0.818]. *Post-hoc* Bonferroni tests revealed that the RTs were significantly longer to phonological targets than to non-phonological targets both in the auditory and visual modality (*p* < 0.001 in both cases). In addition, in the non-phonological tasks, the RTs were significantly shorter to visual targets than to auditory targets (*p* < 0.001).

An ANOVA comparing RTs within the auditory domain indicated a significant effect of Ear [*F*_(1, 25)_ = 7.49, *p* < 0.05, η^2^_*p*_ = 0.230] caused by slightly shorter RTs to the right-ear targets than to the left-ear targets (right ear targets: mean 483.5 ms, s.e.m. 9.5 ms; left ear targets: mean 490.6 ms, s.e.m. 9.7 ms). In addition, there was a significant effect of Task [*F*_(1, 25)_ = 103, *p* < 0.001, η^2^_*p*_ = 0.805] originating from longer RTs to the phonological auditory targets than to the non-phonological auditory targets.

An ANOVA comparing HRs in the auditory and visual tasks indicated a significant effect of Task [*F*_(1, 25)_ = 16.6, *p* < 0.001, η^2^_*p*_ = 0.400] the HRs being lower in the phonological tasks than in the non-phonological tasks. There was also a significant effect of Modality [*F*_(1, 25)_ = 35, *p* < 0.001, η^2^_*p*_ = 0.585] the HRs being higher in the visual tasks than in the auditory tasks.

Comparison of HRs within the auditory domain indicated a significant effect of Ear [*F*_(1, 25)_ = 6.8, *p* < 0.05, η^2^_*p*_ = 0.215]: the HRs were higher in the right-ear tasks than in the left-ear tasks (right ear: mean 96.4%, s.e.m. 0.5%; mean left ear: 95.6%, s.e.m. 0.6%). We also found a significant effect of Task [*F*_(1, 25)_ = 9.3, *p* < 0.01, η^2^_*p*_ = 0.271] due to lower HRs in the A_P_ tasks than in the auditory non-phonological tasks.

Finally, an ANOVA comparing FaRs during the auditory and visual tasks indicated a significant effect of Task [*F*_(1, 25)_ = 48.6, *p* < 0.001, η^2^_*p*_ = 0.661] there being more false alarms during the phonological tasks than during the non-phonological tasks. An ANOVA comparing FaRs in the left-ear and right-ear tasks showed a similar effect of Task [phonological vs. non-phonological; *F*_(1, 25)_ = 31.9, *p* < 0.001, η^2^_*p*_ = 0.561].

### ERPs

#### Effects of attention and task on ERPs to spoken syllables

**Figure 3:**
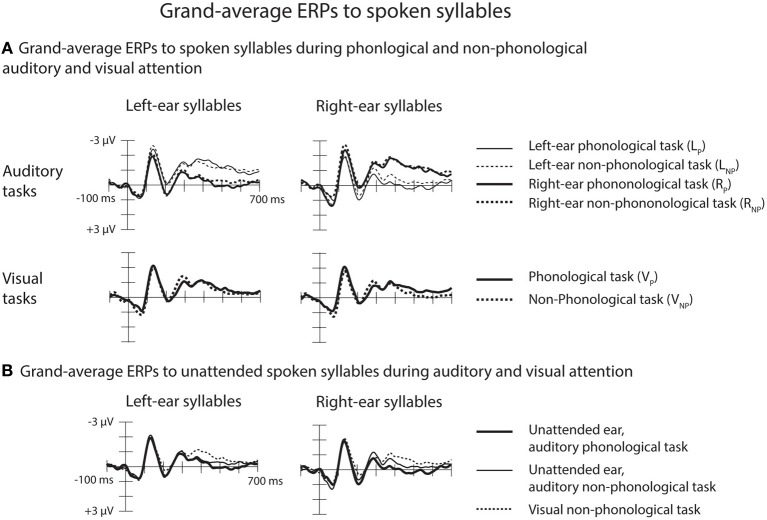
**Grand-average ERPs to spoken syllables. (A)** Top row: Grand-average ERPs at the Cz electrode to the left-ear and right-ear standard syllables during phonological and non-phonological tasks involving the left-ear syllables (L_P_/thin solid lines and L_NP_/thin dotted lines, respectively), during phonological and non-phonological tasks involving the right-ear syllables (R_P_/thick solid lines and R_NP_/thick dotted lines, respectively). Bottom row: Grand-average ERPs at Cz to the left-ear and right-ear standard syllables during visual phonological and non-phonological tasks (V_P_/solid lines and V_NP_/dotted lines, respectively). **(B)** Grand-average ERPs at Cz to unattended left-ear and right-ear standard syllables during phonological (thick solid lines) and non-phonological (thin solid lines) auditory tasks (data combined across the left-ear and right-ear tasks) and during the visual non-phonological task (dotted lines).

**Figure 4:**
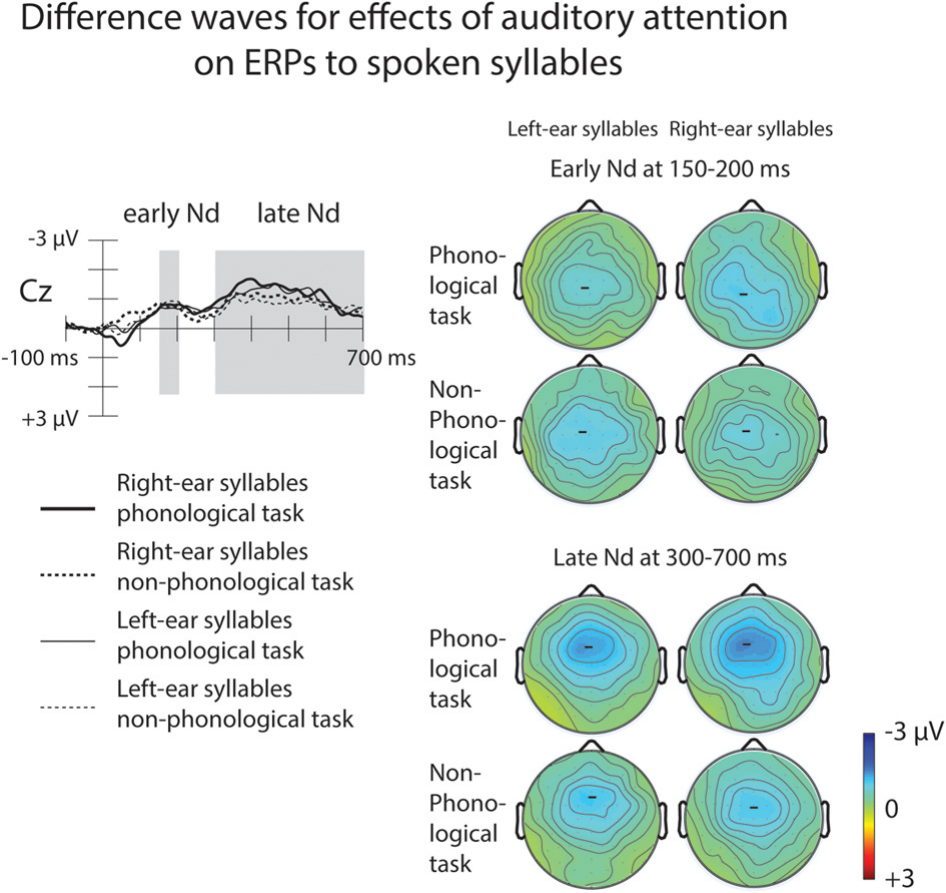
**Effects of auditory attention on ERPs to the left-ear and right-ear syllables illustrated by difference waveforms at the Cz electrode.** The difference waves were obtained by subtracting grand-average ERPs to the left-ear standard syllables during right-ear phonological and non-phonological tasks from grand-average ERPs to the left-ear standard syllables during left-ear phonological and non-phonological tasks, respectively, and by subtracting ERPs to the right-ear standard syllables during left-ear phonological and non-phonological tasks from ERPs to the right-ear standard syllables during right-ear phonological and non-phonological tasks, respectively (for the grand-average ERPs, see Figure 3A, top row). Maps on the right show scalp distributions for the early negative-difference (Nd) effect (150–200 ms from syllable onset) and late Nd effect (300–700 ms from syllable onset) determined after measuring, in each participant, the mean Nd amplitude at each electrode over these time windows marked in the difference waves.

Both for the A_P_ and non-phonological tasks, ERPs to syllables of each ear were negatively displaced when these syllables were attended than when syllables delivered to the other ear were attended (Figure 3A, top row). These Nd effects are seen better in ERP difference waves (Figure 4) obtained separately for each ear and separately for the phonological and non-phonological tasks. These difference waveforms were calculated by subtracting ERPs to the left-ear and right-ear syllables during attention to the syllables to the other ear from ERPs to the same-ear syllables when they were attended. As seen in all these difference waves, the Nd had two phases: an early Nd peaking around 150–200 ms and a late Nd beginning around 250–300 ms from syllable onset and continuing to the end of the analyzed period. The early Nd effect was significant at 150–200 ms and the late Nd effect at 300–700 ms for all auditory conditions (Table 1).

**Table 1:** **Mean amplitudes (μV, standard errors of the mean in parentheses) calculated from difference waveforms (see Figure 4) for the early-Nd and late-Nd effects of auditory attention on ERPs to spoken left-ear and right-ear syllables during phonological and non-phonological tasks at Cz electrode at different time windows from syllable onset**.

**Time window**	**Task**			
	**Phonological task**		**Non-phonological task**	
	**Left-ear syllables**	**Right-ear syllables**	**Left-ear syllables**	**Right-ear syllables**
**EARLY Nd**				
50–100 ms^a^	ns	ns	ns	−0.5 (0.1)^***^
100–150 ms^a^	−0.4 (0.2)^**^	ns	−0.5 (0.1)^***^	−0.7 (0.1)^***^
**150–200 ms^a^**	−0.7 (0.2)^***^	−0.8 (0.2)^***^	−0.9 (0.1)^***^	−0.8 (0.1)^***^
200–250 ms^a^	−0.7 (0.2)^***^	ns	−0.7 (0.2)^**^	−0.5 (0.2)^**^
250–300 ms^a^	−0.6 (0.2)^**^	ns	ns	ns
**LATE Nd**				
**300–400 ms^b^**	−1.1 (0.2)^***^	−1.5 (0.3)^***^	−1.0 (0.2)^***^	−1.0 (0.2)^***^
**400–500 ms^b^**	−1.5 (0.2)^***^	−1.6 (0.2)^***^	−0.9 (0.2)^***^	−1.0 (0.2)^***^
**500–600 ms^b^**	−1.3 (0.2)^***^	−1.5 (0.2)^***^	−1.1 (0.1)^***^	−1.0 (0.2)^***^
**600–700 ms^b^**	−1.0 (0.2)^***^	−1.0 (0.1)^***^	−1.0 (0.1)^***^	−0.7 (0.1)^***^

a Significance from one-sided t-tests Bonferroni corrected for t-tests at the five early-Nd latency windows.

b Significance from one-sided t-tests Bonferroni corrected for t-tests at the four late-Nd latency windows;

*** *p < 0.001*,

** p < 0.01, ns, non-significant. Time windows written in bold font were analyzed further with ANOVAs.

A four-way repeated-measures ANOVA for the early-Nd amplitudes at 150–200 ms revealed a significant Ear × Task × Frontality × Laterality interaction [*F*_(24, 600)_ = 2.03, *p* < 0.05, η^2^_*p*_ = 0.075, ε = 0.334]. The scalp distribution maps (Figure 4) suggested that this interaction was due to a diagonal distribution of the early Nd to right-ear syllables during the phonological tasks. This early Nd was maximal over the left fronto-central and right parietal scalp sites. The early Nd to the left-ear syllables during the phonological task and the early Nds to the left-ear and right-ear syllables during the non-phonological tasks, in turn, showed more symmetric distributions. However, this subtle scalp distribution difference was not verified by further ANOVAs performed separately for each ear, that is, even for the Nds to the right-ear syllables, there was no significant Task × Frontality × Laterality interaction (or Task × Frontality or Task × Laterality interaction).

A four-way repeated-measures ANOVA for the mean late-Nd amplitudes over 300–700 ms revealed a significant Ear × Frontality × Laterality interaction [*F*_(24, 600)_ = 2.59, *p* < 0.01, η^2^_*p*_ = 0.094, ε = 0.333]: for both left-ear and right-ear syllables the late Nd had a fronto-central maximum, but for the right-ear syllables the late Nd spread more to the posterior right-hemisphere electrodes than for the left-ear syllables (Figure 4, bottom right panel).

#### Suppression of the processing of task-irrelevant speech during auditory attention

**Figure 5:**
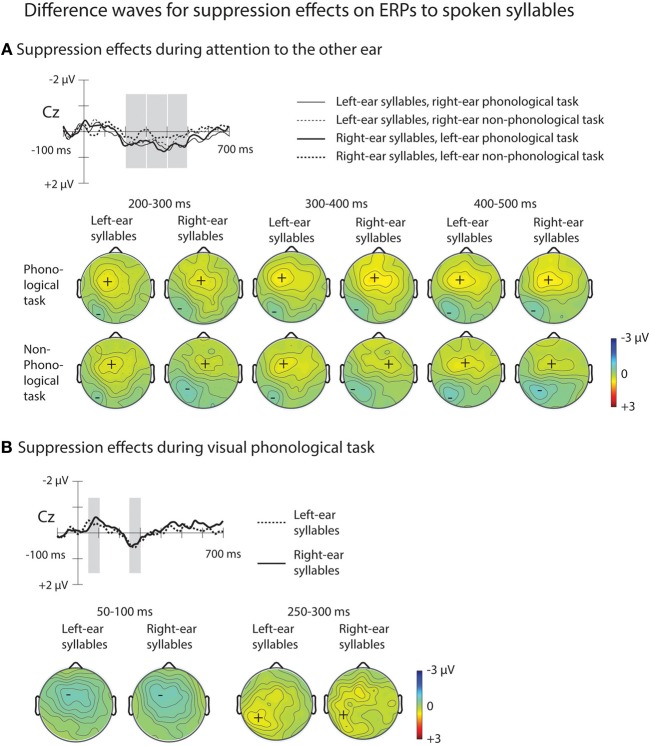
**ERP difference waves illustrating suppression effects on ERPs to spoken syllables (please note that for illustrative purposes, the amplitude scales differ from those used for ERPs in Figures 3, 4). (A)** Difference waves for suppression effects on ERPs to left-ear syllables during the right-ear phonological task at Cz electrode were obtained by subtracting grand-average ERPs to left-ear standard syllables during the visual non-phonological task from grand-average ERPs to the left-ear standard syllables during the right-ear phonological task (thin solid line; for the grand-average ERPs, see Figure 3B). Difference waves for suppression effects on ERPs to the right-ear syllables during the left-ear phonological tasks were obtained by subtracting grand-average ERPs to the right-ear syllables during the visual non-phonological task from grand-average ERPs to the right-ear syllables during the left-ear phonological task (thick solid line). Difference waveforms for non-phonological tasks were obtained accordingly (thin dotted line for left-ear syllables and thick dotted line for right-ear syllables during non-phonological tasks to the other ear, respectively). Maps below these difference waves show scalp distributions of suppression effects (the rejection positivity; RP) measured in each participant at each electrode over the time windows (marked in Cz difference waves) 200–300, 300–400, 400–500 ms from syllable onset. **(B)** Difference waves at Cz electrode illustrating suppression effects on ERPs to spoken syllables during the visual phonological task. Difference waves for the left-ear and right-ear standard syllables (dotted and solid line, respectively) were obtained by subtracting grand-average ERPs to the left-ear and right-ear syllables during the visual non-phonological task from grand-average ERPs to the left-ear and right-ear standard syllables, respectively, during the visual phonological task (for the grand-average ERPs, see Figure 3A, bottom row). The analysis windows at 250–300 ms for the rejection positivity (RP) and at 50–100 ms for the preceding negative ERP displacement are marked with gray rectangles in the difference waves at the Cz electrode and maps for the scalp distribution of the mean amplitudes at these time windows are shown below separately for the left-ear and right-ear syllables.

As expected, the ERPs for syllables of each ear were positively displaced when syllables to the other ear were attended during the phonological or non-phonological task than when spoken syllables were ignored during the visual tasks (Figure 3B). These RPs are better seen in suppression difference waveforms (Figure 5A) obtained by subtracting ERPs to the left-ear and right-ear standard syllables during the V_NP_ task from ERPs to these syllables during the R_P_ and R_NP_ tasks. We expected to find positive displacements of ERPs to the syllables delivered to one ear during attention to the syllables delivered to the other ear in relation to ERPs to the syllables during the V_NP_ task around 200–300 ms from syllable onset (cf. Alho et al., 1994) Indeed, the RPs were significant at Cz electrode at 200–250 and 250–300 ms in all cases except for the right-ear syllables during the L_P_ task (Table 2A). Figure 5A shows scalp distributions of these RPs at 200–300 ms. However, as seen in Figure 5A, the RPs continued longer than expected and, therefore, scalp distribution maps are shown also for two consecutive 100-ms time windows. Hence, similar ANOVAs were calculated for the 300–400 and 400–500 ms time windows as for the 200–300 ms window.

**Table 2:** **Mean rejection-positivity (RP) amplitudes (μV; standard errors of the mean in parentheses) calculated from ERP difference waveforms (see Figure 5) at the Cz electrode**.

	**Left-ear syllables**		**Right-ear syllables**	
**Time window**	**Right-ear phonological task**	**Right-ear non-phonological task**	**Left-ear phonological task**	**Left-ear non-phonological task**
**(A) SUPPRESSION EFFECTS ON AUDITORY SYLLABLES DURING AUDITORY TASKS**				
150–200 ms^a^	ns	ns	ns	ns
**200–250 ms**^a^	0.5 (0.2)^**^	0.5 (0.2)^**^	0.5 (0.2)^**^	ns
**250–300 ms**^a^	0.5 (0.2)^**^	0.4 (0.2)^*^	0.4 (0.2)^**^	ns
**(B) SUPPRESSION EFFECTS ON SPOKEN SYLLABLES DURING THE VISUAL PHONOLOGICAL TASK**				
150–200 ms^a^	ns		ns	
200–250 ms^a^	ns		ns	
**250–300 ms**^a^	0.5 (0.1)^**^		ns	

RPs were obtained by subtracting ERPs to the left-ear and right-ear syllables during the visual non-phonological (V_NP_) task from ERPs to the left-ear and right-ear syllables, respectively, that were to be ignored during phonological or non-phonological task involving the opposite-ear syllables (R_P_/R_NP_ and L_P_/L_NP_, respectively) or during the visual phonological (V_P_) task.

**(A)** RP in ERPs to spoken syllables delivered to one ear during attention to the opposite ear and **(B)** RP in ERPs to spoken syllables during the V_P_ task at Cz electrode are reported if statistically significant from zero.

a Significance from one-sided t-tests Bonferroni corrected for t-tests at the 3 predetermined time windows.

** *p < 0.01*,

* p < 0.05, ns, non-significant. Time windows written in bold font were analyzed further with ANOVAs.

A Four-Way ANOVA on the mean RP amplitudes at 200–300 ms yielded a significant Ear × Laterality interaction [*F*_(6, 150)_ = 4.42, *p* < 0.05, η^2^_*p*_ = 0.150, ε = 0.390]. This was due to less RP over the left hemisphere for unattended right-ear syllables than for unattended left-ear syllables during attention to the opposite-ear syllables. In addition, a Four-Way ANOVA at 300–400 ms yielded a significant main effect of Task [*F*_(1, 25)_ = 5.88, *p* < 0.05, η^2^_*p*_ = 0.191] with higher RP amplitudes during the phonological auditory tasks than during the non-phonological auditory tasks. At 400–500 ms, there was a significant Task × Laterality interaction [*F*_(6, 150)_ = 3.57, *p* < 0.05, η^2^_*p*_ = 0.125, ε = 0.404] with higher RP amplitudes during the phonological tasks than during the non-phonological tasks, an effect that was stronger over the left hemisphere than over the right hemisphere.

#### Suppression of task-irrelevant speech during visual attention

As predicted, an RP was also observed for unattended spoken syllables during the V_P_ task: ERPs to the left-ear and right-ear syllables were positively displaced during the V_P_ task in relation to the V_NP_ task around 200–300 ms from syllable onset (Figure 3A, bottom row). These effects are seen in suppression difference waveforms obtained by subtracting ERPs to the left-ear and right-ear standard syllables during the V_NP_ task from ERPs to these syllables during the V_P_ task (Figure 5B; Table 2B). As seen in Table 2B, the RP for the V_P_ task was significant in ERPs to unattended left-ear syllables but not in ERPs to unattended right-ear syllables. A repeated-measure ANOVA for the RP amplitudes at 250–300 ms showed a significant Frontality × Laterality interaction [*F*_(24, 600)_ = 3.49, *p* < 0.001, η^2^_*p*_ = 0.122, ε = 0.296]. As seen in scalp distribution maps (Figure 5B), the RP was larger over the left hemisphere than over the right hemisphere independent of which ear was stimulated.

As seen in Figure 5B, the RPs were unexpectedly preceded by more negative ERPs around 100 ms to the unattended spoken syllables during the V_P_ task than during the V_NP_ task. The amplitudes of these effects were measured as mean amplitudes of the ERP difference waves at Cz (shown in Figure 5B) over 50–100 and 100–150 ms from syllable onset. For both left-ear and right-ear syllables, the amplitude differed significantly from 0 μV at 50–100 ms (left-ear syllables: *t*_(26)_ = −2.26, *p* < 0.05; right-ear syllables: *t*_(26)_ = −2.39, *p* < 0.05; significance corrected for comparisons at two time windows), but not at 100–150 ms [left-ear syllables: *t*_(26)_ = −2.26, *p* < 0.05; right-ear syllables: *t*_(26)_ = −2.39, *p* < 0.05]. A subsequent repeated-measures ANOVA for the amplitude of this negativity at 50–100 ms with factors Stimulated Ear, Frontality, and Laterality showed a significant effect of Frontality [*F*_(4, 96)_ = 6.40, *p* < 0.05, η^2^_*p*_ = 0.211, ε = 0.331] and Laterality [*F*_(6, 144)_ = 3.77, *p* < 0.05, η^2^_*p*_ = 0.136, ε = 0.390], as well as a significant Frontality × Laterality interaction [*F*_(24, 576)_ = 2.16, *p* < 0.05, η^2^_*p*_ = 0.083, ε = 0.291]. This was due to a larger negativity over the left frontal scalp areas than over the right frontal scalp areas for both the left-ear and right-ear syllables.

A repeated-measures ANOVA comparing the RP amplitude at 200–300 ms to unattended left-ear and right-ear syllables during the R_P_ and L_P_ tasks, respectively (Figure 5A) with the RP at 250–300 ms to unattended syllables during the V_P_ task (Figure 5B) yielded a significant Attended Modality × Frontality × Laterality interaction [*F*_(24, 600)_ = 6.11, *p* < 0.001, η^2^_*p*_ = 0.196, ε = 0.306]: the RP to unattended syllables was larger over the left-hemisphere centro-parietal scalp during the V_P_ task than during the A_P_ tasks involving the opposite-ear syllables.

#### Effects of attention and task on ERPs to written letters

**Figure 6:**
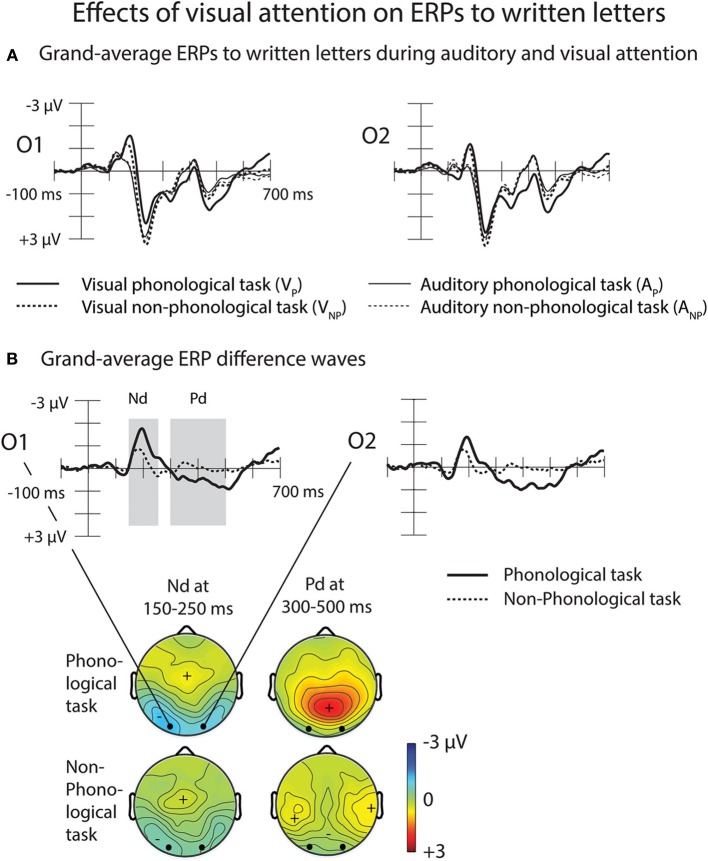
**Effects of visual attention on ERPs to written letters. (A)** Grand-average ERPs to written letters during auditory and visual attention at the O1 and O2 electrodes. **(B)** Effects of visual attention on ERPs to written letters at the O1 and the O2 electrodes illustrated by difference waves obtained by subtracting grand-average visual ERPs during auditory phonological tasks (solid lines, data combined across the left-ear and right-ear phonological tasks) and during auditory non-phonological tasks (dotted lines, data combined across the left-ear and right-ear non-phonological tasks) from grand-average visual ERPs during the visual phonological and visual non-phonological tasks, respectively. Scalp distribution maps on the right panel show the visual Nd effect (150–250 ms from letter appearance) and the subsequent Pd effect (300–500 ms from letter appearance) determined after measuring in each participant the mean Nd and Pd amplitudes at each electrode over these time windows marked in the O1 and O2 difference waves. Please note that the negative Nd maxima are shown in cyan/blue and positive Pd maxima in yellow/red.

Grand-average ERPs to written letters during auditory and visual attention are illustrated in Figure 6A. To examine effects of attention on letter processing, difference waves were calculated by subtracting ERPs to the visual standard stimuli during the A_P_ tasks (data collapsed across the L_P_ and R_P_ tasks) from ERPs to the visual standard stimuli during the V_P_ task (for grand-average difference waves, see Figure 6B). Difference waveforms for the non-phonological tasks were calculated accordingly. The difference waves for both V_P_ and V_NP_ tasks had two phases, an early Nd peaking around 150–250 ms and a later Pd peaking around 300–600 ms from letter appearance. We observed significant Nd and Pd effects at latency ranges 150–250 and 300–500 ms from letter appearance, respectively (Table 3). These time windows were selected for further analysis (for scalp distribution maps of the Nd at 150–250 ms and Pd at 300–500 ms, see the right panel of Figure 6A).

**Table 3:** **Mean amplitudes (μV, standard errors of the mean in parentheses) and their significant difference from 0 μV of the Nd and Pd effects of visual attention on ERPs to written letters at different time windows from appearance of letter**.

	**Phonological tasks**	**Non-phonological tasks**
**150–200 ms^a^**	−0.9 (0.3)^***^	−0.6 (0.1)^***^
**200–250 ms^a^**	−0.8 (0.4)^*^	ns
250–300 ms^a^	ns	ns
**300–400 ms^a^**	−0.6 (0.2)^**^	ns
**400–500 ms^a^**	−0.8 (0.2)^**^	ns
500–600 ms^a^	ns	ns

The amplitudes were measured from collapsed O1/O2 difference waveforms (see Figure 6) obtained by subtracting ERPs to the visual standard stimuli during the A_P_ tasks from ERPs to the visual standard stimuli during the V_P_ task and by subtracting ERPs to the visual standard stimuli during the A_NP_ tasks from ERPs to the visual standard stimuli during the V_NP_ task.

a Significance from one-sided t-tests Bonferroni corrected for t-tests at 3 latency windows.

*** *p < 0.001*,

** *p < 0.01*,

* p < 0.05, ns, non-significant. Time windows written in bold font were analyzed further with ANOVAs.

A Three-Way ANOVA for the visual-Nd time window showed a significant Task × Frontality interaction [*F*_(5, 125)_ = 8.55, *p* < 0.005, η^2^_*p*_ = 0.255, ε = 0.300]. Visual-Nd amplitudes were larger at occipital sites during phonological than during non-phonological conditions (Figure 6A, right panel). For the Pd effect, a Three-Way ANOVA revealed a significant Task × Laterality × Frontality interaction [*F*_(10, 250)_ = 5.581, *p* < 0.001, η^2^_*p*_ = 0.183, ε = 0.522], caused by larger Pd amplitudes at all electrode sites, especially over the central to occipital sites during phonological conditions than non-phonological conditions.

**Figure 7:**
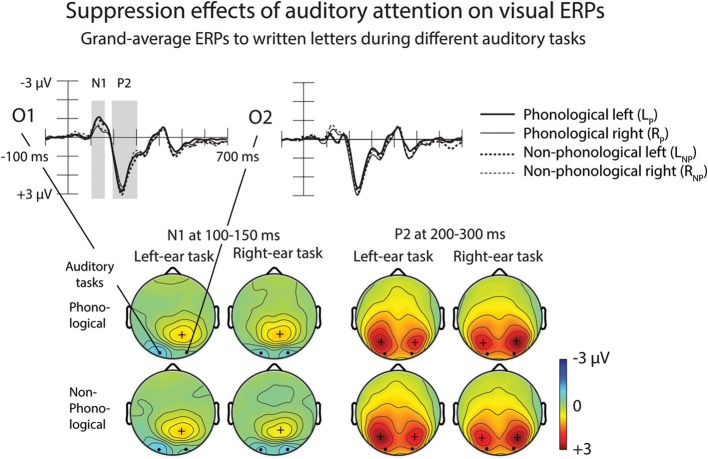
**Suppression effects of auditory attention on visual ERPs.** Grand-average ERPs to written letters during different auditory tasks at the O1 and O2 electrodes are shown on the upper panel. Scalp maps on the lower panel show N1 and P2 responses at 100–150 and 200–300 ms from letter appearance, respectively. Time windows are marked in the O1 and O2 difference waves. Please note that the negative N1 maxima are shown on cyan/blue and positive P2 maxima in red.

Figure 7 illustrates effects of auditory tasks on N1 and P2 responses to written letters. Amplitudes of the N1 and P2 responses to letters during the four auditory tasks were measured at O1 and O2 as mean amplitudes over 100–150 and 200–300 ms from letter appearance (Table 4). A Three-Way ANOVA for the visual N1 amplitudes during the auditory L_P_, L_NP_, R_P_, and R_NP_ tasks showed a significant Attended Ear × Electrode interaction [*F*_(1, 25)_ = 12.39, *p* < 0.005, η^2^_*p*_ = 0.331]. The N1 responses to the written letters were larger during attention to the left-ear syllables than during attention to the right-ear syllables at O1 electrode over the left visual cortex (*p* < 0.05, Bonferroni-corrected), while an opposite effect was found at O2 over the right visual cortex (*p* < 0.01, Bonferroni-corrected). In addition, during left-ear attention, the visual N1 responses were larger at O1 than at O2 (*p* < 0.005, Bonferroni-corrected), whereas there were no such significant difference during right-ear attention. Similar effects were observed for the visual P2 [Attended Ear × Electrode interaction: *F*_(1, 25)_ = 17.123, *p* < 0.001, η^2^_*p*_ = 0.407]: the P2 responses to the written letters were larger during left-ear attention than during right-ear attention at O1 (*p* < 0.05, Bonferroni-corrected), and larger during right-ear attention than during left-ear attention at O2 (*p* < 0.05, Bonferroni-corrected). During left-ear attention, the P2 responses were larger at O1 than at O2 (*p* < 0.05, Bonferroni-corrected) whereas there were no such significant differences during right-ear attention. For both visual N1 and P2 responses, no significant differences were observed between the A_P_ and non-phonological conditions.

**Table 4:** **Mean amplitudes (μV, standard errors of the mean in parentheses) of N1 and P2 responses to to-be-ignore written letters, measured at O1 and O2 electrodes over time windows 100–150 ms and 200–300 ms from letter appearance, respectively, during auditory left-ear and right-ear phonological and non-phonological tasks**.

	**Phonological task**		**Non-phonological task**	
	**Left-ear task**	**Right-ear task**	**Left-ear task**	**Right-ear task**
**O1 ELECTRODE**				
N1 (100–150 ms)	−0.3 (0.4)	−0.1 (0.3)	−0.3 (0.4)	−0.0 (0.4)
P2 (200–300 ms)	2.0 (0.3)	1.9 (0.3)	2.2 (0.4)	1.9 (0.3)
**O2 ELECTRODE**				
N1 (100–150 ms)	−0.1 (0.3)	−0.0 (0.3)	−0.1 (0.3)	−0.2 (0.3)
P2 (200–300 ms)	1.7 (0.3)	2.1 (0.3)	1.8 (0.3)	1.9 (0.3)

## Discussion

### Effects of auditory attention on the processing of task-relevant speech

As expected, the ERPs for selectively attended spoken syllables were negatively displaced in relation to the ERPs to unattended syllables with an early Nd at 150–200 ms and a late Nd at 300–700 ms (Figure 4). There was a significant but subtle scalp distribution difference between the early Nd obtained for the right-ear phonological task and the early Nds for the left-ear phonological task and right-ear and left-ear non-phonological tasks. However, we found no systematic effect of the auditory task on the overall amplitude of the early (or late) Nd, suggesting that the Nd indeed indexes selection of relevant auditory stimuli for further processing and is insensitive to the type of this processing.

### Suppression of task-irrelevant speech during auditory and visual attention

In the present study, the ERPs to spoken syllables were positively displaced when syllables to the other ear were attended to in relation to the ERPs to the same syllables during the visual non-phonological task (Figure 5A). This result is consistent with previous studies reporting a RP to unattended non-speech sounds and suggesting active suppression of task-irrelevant sounds during selective listening to other sounds (Alho et al., 1987, 1994; Berman et al., 1989; Michie et al., 1990; Woods, 1990; Degerman et al., 2008). The present RPs to syllables unattended during attention to the other ear were affected by the stimulated ear: a smaller RP was found over the language-dominant left hemisphere for the unattended right-ear syllables than for the unattended left-ear syllables, while there was no effect of stimulated ear on the RP measured over the right hemisphere. Perhaps due to REA, it was more difficult to suppress the processing of right-ear syllables than the processing of the left-ear syllables (Kinsbourne, 1970; Takio et al., 2011; Alho et al., 2012). Occurrence of REA in the present experimental paradigm was suggested by higher HRs and slightly shorter RTs for the right-ear targets than for the left-ear targets. Furthermore, at 300–400 ms we found larger RP amplitudes to unattended syllables during the A_P_ tasks than during the auditory non-phonological tasks and at 400–500 ms, the RP was lateralized to the left hemisphere. These findings suggest overall delayed suppression of irrelevant speech during phonological processing of other speech stimuli and that this suppression occurs predominantly in the language-dominant left hemisphere.

Moreover, we found RPs in ERPs to spoken syllables ignored during the visual phonological task in relation to ERPs to the same syllables during the visual non-phonological task. This result was expected due to our previous fMRI results (Salo et al., 2013) indicating suppression of irrelevant speech during visual phonological processing. These RPs suggest that there was a stronger need to ignore the distracting irrelevant speech sounds during this task than during the visual non-phonological task. These RPs were larger over the language-dominant left hemisphere than over the right hemisphere at 250–300 ms, independent of the stimulated ear (Figure 5B). This is consistent with the fMRI results of Salo et al. (2013) indicating suppression of task-irrelevant speech stimuli during the visual phonological task especially in the left auditory cortex. Unexpectedly the RP to syllables during the visual phonological task was preceded by an early left-hemisphere dominant negative displacement in ERPs to these syllables in relation to the ERPs to the syllables during the visual non-phonological task. This suggests that during visual phonological processing (e.g., reading) irrelevant speech indeed intrudes to the left-hemisphere speech processing systems. Perhaps phonological visual processing automatically facilitates A_P_ processing and leads to this intrusion which would be followed by a need to actively suppress the processing of irrelevant speech as reflected by the RP.

Moreover, comparison of RPs to unattended spoken syllables during the visual phonological task with those during the A_P_ tasks indicated that RPs were larger over the left hemisphere during the visual phonological task than during the A_P_ tasks. This suggests strong suppression of speech processing in the language-dominant hemisphere during phonological processing of written text. One reason for this could be that reading letters is an ability acquired later in life whereas extracting linguistic information from hearing develops already in infancy. Perhaps therefore task-irrelevant speech distracts more easily reading than listening to other speech leading to a stronger need for suppression of irrelevant speech during reading.

### Effects of visual attention and task on ERPs to written letters

Consistent with our hypothesis and previous results (e.g., Hillyard and Anllo-Vento, 1998; Salmi et al., 2007), we found a visual early Nd peaking around 150–250 ms at occipital sites in the ERPs to letters during the visual tasks in relation to the auditory tasks (Figure 6). This Nd was larger for the phonological visual task than for the non-phonological visual task. Moreover, we found a subsequent Pd effect at 300–500 ms that was also larger over the posterior scalp during phonological than non-phonological tasks (Figure 6). These results suggest that there may have been a higher demand for attention in the more difficult visual phonological task than in the visual non-phonological task.

### Modulation of the processing of task-irrelevant letters by auditory attention

During the four auditory tasks, the N1 and P2 to unattended written letters were larger at the O1 electrode during attention to the left-ear syllables than during attention to the right-ear syllables, whereas the opposite was true for the O2 electrode (Figure 7). One would, however, expect foveal letters to elicit N1 and P2 that are distributed symmetrically over the left and right posterior scalp (see, e.g., Vogel and Luck, 2000). In the present study, such symmetrical distributions were observed for the N1 and P2 responses to the unattended letters during attention to the right ear. Therefore, the difference in the laterality of N1 and P2 responses to unattended foveal letters between conditions demanding attention to the left ear and conditions demanding attention to the right ear was presumably due to an effect of left-ear attention on processing of unattended letters. Perhaps the processing of task-irrelevant letters was suppressed in the visual cortex of the contralateral hemisphere and enhanced in the ipsilateral visual cortex especially during the left-ear tasks that were, according to the HRs and RTs, more difficult than the right-ear tasks. This finding parallels Salo et al.'s (2013) tentative fMRI results of possible suppression of the processing of task-irrelevant letters in the right occipital cortex during phonological and non-phonological tasks involving binaural spoken syllables.

### Conclusions

Consistent with previous ERP results, we found Nd effects associated with selective attention to spoken syllables. Moreover, we observed attention-related Nd and Pd attention effects in ERPs to written letters. Active suppression of task-irrelevant speech, in turn, was reflected by RP responses to task-irrelevant spoken syllables during the A_P_ and non-phonological tasks, as well as during the visual phonological task. Finally, we observed that N1 and P2 responses to unattended written letters were attenuated in the left hemisphere and augmented in the right hemisphere during left-ear phonological and non-phonological tasks. This result suggests that demanding auditory tasks may modulate the processing of task-irrelevant visual stimuli.

### Conflict of interest statement

The authors declare that the research was conducted in the absence of any commercial or financial relationships that could be construed as a potential conflict of interest.
